# Prevalence of insomnia in nurses: a meta-analysis of observational studies using the insomnia severity index

**DOI:** 10.3389/fpubh.2026.1913239

**Published:** 2026-08-12

**Authors:** Yunpeng Wu, Tao Cheng, Luying Zhong, Changqi Ye

**Affiliations:** 1 Emergency Department of West China Hospital, Sichuan University/West China School of Nursing, Sichuan University, Chengdu, China; 2 Disaster Medical Center, Sichuan University, Chengdu, China; 3 Nursing Key Laboratory of Sichuan Province, Chengdu, China

**Keywords:** meta-analysis, nurses, occupational health, prevalence, sleep initiation and maintenance disorders

## Abstract

**Background:**

Although the prevalence of insomnia in nurses has been extensively studied, reported estimates vary considerably across studies. This meta-analysis of observational studies was conducted to determine the pooled prevalence of insomnia and to explore its potential moderators in nurses.

**Methods:**

A computerized literature search was conducted in PubMed, Web of Science, Scopus, Embase, Cochrane Library, CINAHL, and PsycINFO from inception to June 2026. A random-effects model was used to generate pooled prevalence estimates, along with 95% confidence intervals and prediction intervals. Heterogeneity was assessed using the I^2^ statistic, and subgroup analyses and meta-regression were performed to identify its potential moderators.

**Results:**

Based on 51 studies from 19 countries encompassing 36,971 participants, the pooled prevalence of insomnia in nurses was 55.0% (95% CI: 49.2–60.8, 95% prediction interval: 15.8–90.8%, I^2^ = 99.1%). Subgroup analyses and meta-regression demonstrated that work unit was a significant moderator of the pooled prevalence (*p* < 0.001).

**Conclusion:**

Our findings suggest that over half of nurses experience insomnia, though considerable heterogeneity warrants caution in interpretation. Moreover, work unit was identified as a significant moderator. These findings highlight the urgent need for routine sleep screening and evidence-based interventions to address the high insomnia burden in this occupational group.

## 1 Introduction

As frontline healthcare professionals, nurses provide direct clinical care through the monitoring of disease progression, health guidance, and psychological counseling, making them irreplaceable in safeguarding healthcare quality and patient safety (1). However, nurses are generally confronted with occupational stressors such as shift work, high-intensity tasks, and exposure to patient suffering and mortality (2). The chronic accumulation of these stressors not only consumes nurses’ physical and psychological resources but also renders them vulnerable to a variety of health impairments, including cardiovascular diseases, metabolic disorders, anxiety, and depression (3, 4). In particular, insomnia has emerged as a major concern owing to its high prevalence and persistent underrecognition, and has increasingly become a priority area in nursing occupational health research (5).

Insomnia is a highly prevalent and burdensome sleep disorder, characterized by difficulty with sleep initiation, maintenance, or early awakening, along with daytime impairments including fatigue, inattention, mood disturbance, and decreased social and occupational functioning (6). For nurses, frequent night shift rotations directly disrupt circadian rhythms and interfere with normal melatonin secretion. The sustained vigilance demanded during shifts keeps the sympathetic nervous system chronically activated, hindering physical and mental relaxation even after work, and compromising sleep quality (7, 8). A growing body of literature has demonstrated that insomnia in nurses is associated with adverse health outcomes, including cardiovascular disease, metabolic disorders, depression, and anxiety, and occupational impairments, such as medical errors, diminished clinical judgment, and impaired emergency response, which significantly threaten patient safety and care quality (9–11). Furthermore, insomnia has been implicated in a range of organizational-level detrimental outcomes, encompassing burnout, reduced occupational satisfaction, and elevated intentions to leave, which may further exacerbate the depletion of the nursing workforce (12–14). Given these considerations, a comprehensive assessment of the prevalence of insomnia in nurses is critical for the development of evidence-informed occupational health policies.

The Insomnia Severity Index (ISI), characterized by robust psychometric properties, concise item content, and comprehensive evaluation of both nocturnal symptom severity and daytime functional impairment, has become one of the most well-established self-report instruments for assessing insomnia in nurses (15). Many studies have employed the ISI to determine the prevalence of insomnia among nurses in diverse cultural settings, including intensive care units, outpatient department, hemodialysis centers, and multi-departmental nursing samples (16–19). However, the reported prevalence estimates have exhibited considerable variation, ranging from 8.2% to 98.9% (16–19). The discrepancies may be attributable to a range of factors, including economic status, cultural context, clinical specialty, age distribution, and timing of data collection. This considerable heterogeneity not only impedes the establishment of a robust and reliable pooled prevalence estimate for insomnia in nurses but also constrains meaningful comparisons across individual studies. Notably, while the ISI is widely used to screen for insomnia, it is essential to distinguish between general sleep disturbances, such as transient difficulties in initiating or maintaining sleep, and clinically defined insomnia. The latter requires both night-time symptoms and clinically significant daytime impairment or distress, as operationalized by the ISI’s composite scoring algorithm. Failure to differentiate these constructs may lead to overestimation of true insomnia prevalence and obscure clinical interpretability. Moreover, the sources of heterogeneity across studies are often discussed superficially, without clarifying the underlying mechanisms of moderator variables. For instance, cultural norms may shape subjective thresholds for reporting sleep complaints. Economic conditions may influence work schedules and environmental stressors. Specialty-specific workloads may differentially affect the severity and chronicity of sleep disturbances. Without clear articulation of these pathways, the interpretation of heterogeneity remains speculative rather than explanatory. To date, although several meta-analyses have examined sleep quality or sleep disturbances among nurses (20, 21), no study has specifically synthesized the prevalence of insomnia in nurses using the ISI. This gap limits accurate burden estimation of insomnia in nurses and the development of empirical screening protocols and intervention frameworks by healthcare decision-makers. Therefore, this meta-analysis of observational studies using the ISI aimed to determine the pooled prevalence of insomnia and identify its potential moderating factors in nurses.

## 2 Methods

This meta-analysis was conducted in accordance with the PRISMA guidelines (22). The study protocol was prospectively registered with PROSPERO (CRD420261425169).

### 2.1 Search strategy

A computerized literature search of PubMed, Web of Science, Scopus, Embase, Cochrane Library, CINAHL, and PsycINFO was conducted from inception to June 2026, restricted to articles published in English (Supplementary Table S1). Boolean logic was applied to combine MeSH terms and corresponding free keywords, including nurses, nursing staff, sleep initiation and maintenance disorders, insomnia, Insomnia Severity Index, and ISI, in the search strategy. In addition, reference lists of all included studies were searched by hand to identify further eligible articles.

### 2.2 Inclusion and exclusion criteria

Studies were eligible if they: (1) enrolled clinical nurses as participants; (2) used a cross-sectional or cohort design; (3) assessed insomnia using the ISI with a cut-off of 8; (4) provided prevalence data of insomnia or sufficient information for its computation. For cohort studies, only baseline prevalence data were extracted and follow-up or incidence data were not included in the analysis. Case–control studies were excluded because prevalence estimates derived from such designs are contingent upon the investigator-specified case-to-control ratio, which does not reflect the true population distribution. Conference abstracts, study protocols, case reports, reviews, and duplicate publications were excluded, as were non-English articles and grey literature, including dissertations and non-peer-reviewed papers.

### 2.3 Screening and data extraction

Two reviewers independently performed study selection and data extraction. For study selection, titles and abstracts were first screened, followed by full-text review of potentially eligible articles. Retrieved records were managed using EndNote for deduplication and organization. For data extraction, a standardized data collection form was used by the two reviewers independently. Any disagreements at either stage were resolved through discussion or consultation with a third reviewer. The extracted information included author, publication year, country, study design, sample size, mean age, proportions of females and married participants, work unit, and insomnia prevalence. When critical data were missing, we contacted the corresponding authors by email.

### 2.4 Risk of bias assessment

The risk of bias of each study was independently assessed by two reviewers using the JBI critical appraisal checklist for prevalence studies (23), which covers nine domains. For each domain, ratings of “yes,” “no,” or “unclear/not applicable” were assigned. Based on the proportion of “yes” responses, studies were classified as low (≥ 70%), moderate (50–69%), or high (≤49%) risk of bias. Any disagreement between reviewers was resolved by consulting a third reviewer.

### 2.5 Statistical analysis

Statistical analyses were performed with Stata 19.0. Before the main meta-analysis, we applied the Freeman-Tukey double arcsine transformation to normalize the distribution and stabilize the variance of the prevalence estimates. This is a necessary step for bounded proportion data. This transformation was chosen over the logit alternative because its variance depends solely on the sample size, not on random event counts, thereby avoiding intrinsic correlation between the transformed estimate and its variance and obviating the need for continuity corrections when zero events occur. Heterogeneity was evaluated using the I^2^ statistic, with ≥ 50% indicating substantial heterogeneity. A random-effects model employing restricted maximum likelihood (REML) estimation was then employed to estimate the pooled prevalence and its 95% confidence interval (CI). Moreover, the 95% prediction interval (PI) was also computed to provide the expected range of true prevalence in future investigations. Subgroup analyses were conducted by economic condition, geographic region, data collection period, and work unit to investigate potential heterogeneity. In addition, univariate meta-regression was conducted to assess the relationship between the pooled prevalence and covariates, including publication year, sample size, mean age, proportions of females and married participants, as well as the subgroup variables. Publication bias was assessed using funnel plots and Egger’s test. Robustness of findings was tested via a leave-one-out sensitivity analysis.

## 3 Results

### 3.1 Literature screening and characteristics

**Figure 1:**
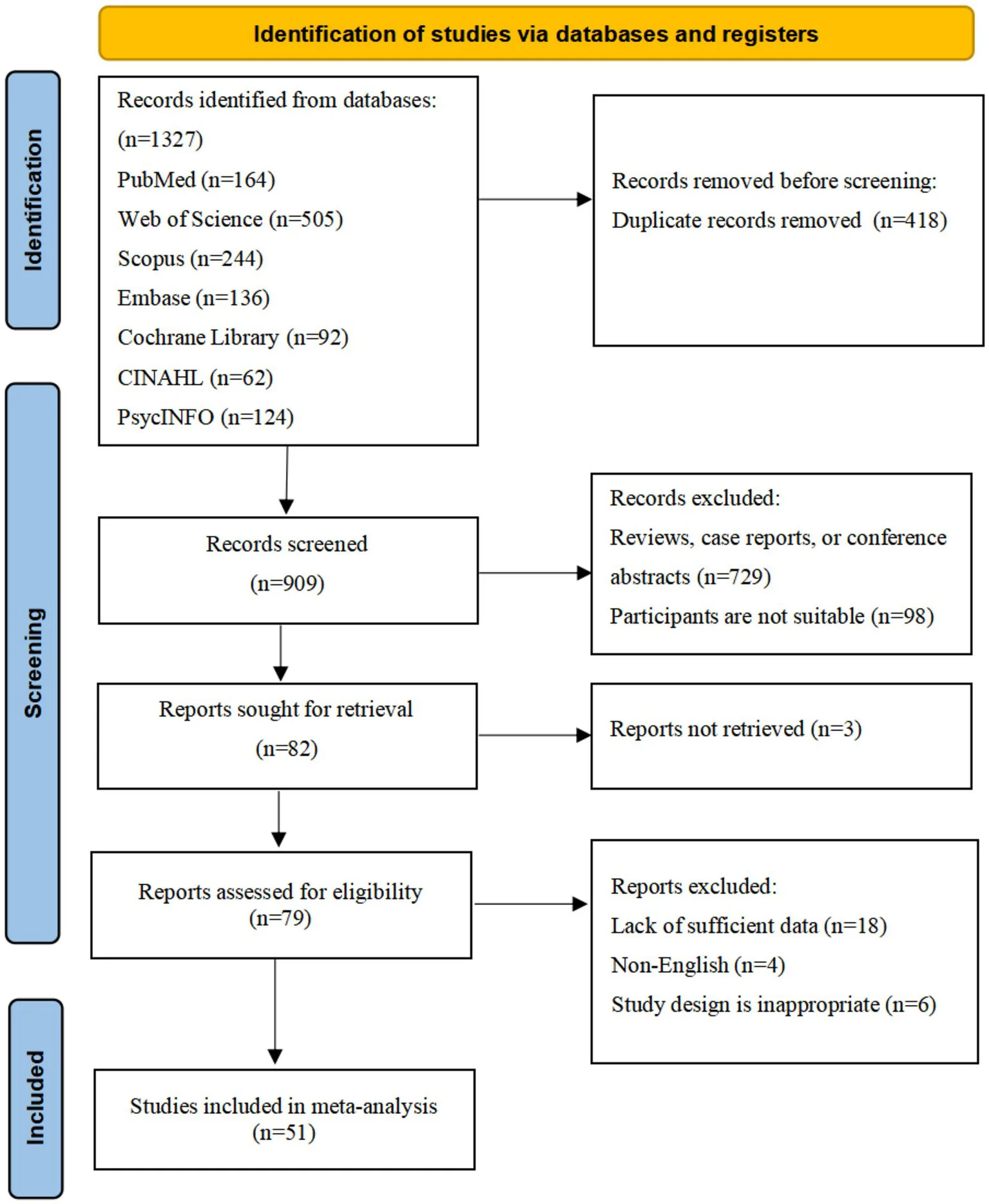
Flow diagram of study selection.

The initial database search yielded a total of 1,327 citations. After deduplication, 418 records were removed, leaving 909 studies for title and abstract assessment. Screening at this stage excluded a further 727 records, resulting in 79 articles selected for full-text review. A subsequent detailed evaluation led to the exclusion of 28 articles, primarily due to incomplete data, non-English text, or methodological concerns. Consequently, a final set of 51 studies met the predefined eligibility criteria and were included in the quantitative synthesis. The complete screening workflow is illustrated in Figure 1. Regarding study design, the majority of the included studies employed a cross-sectional design, with only two providing baseline data from cohort studies. These studies were conducted in 19 countries, with the majority located in Asia. Furthermore, the majority of the included studies enrolled participants from various departments within the respective healthcare settings, with sample sizes ranging from 84 to 3,378 participants. The prevalence of insomnia reported across the included studies varied considerably, with a range of 8.2 to 98.9%. The study characteristics are provided in Table 1.

**Table 1:** Characteristics of included studies.

Study	Country	Study design	Sample size	Married (%)	Mean age (years)	Female (%)	Work unit	Prevalence (%)
Al Nabhani et al. (35)	Oman	Cross-sectional	271	92.3	NA	86.7	Multiple departments	57.6
Alhurishi et al. (36)	Saudi Arabia	Cross-sectional	147	NA	NA	NA	Multiple departments	63.9
Ali et al. (36)	Kenya	Cross-sectional	171	60.8	33.5	70.2	Multiple departments	36.3
Bazrafshan et al. (37)	Iran	Cross-sectional	100	57	30.4	49	Multiple departments	55
Bennaroch and Shochat (26)	Israel	Cross-sectional	448	73.7	41.7	100	Multiple departments	70.0
Chen et al. (38)	China	Cross-sectional	1,812	83	38	88.6	Geriatrics department	49.4
Chen et al. (39)	China	Cross-sectional	501	62.5	NA	93.2	Multiple departments	51.3
Chung et al. (32)	Korea	Cross-sectional	386	NA	32.6	NA	Multiple departments	44.3
Cici and Yilmazel (40)	Turkey	Cross-sectional	2,930	57.7	31.8	77.8	Multiple departments	69.6
Da’seh et al. (41)	Jordan	Cross-sectional	364	39.8	30	62.4	Multiple departments	67.3
Giménez-Díez et al. (42)	Spain	Cross-sectional	1,051	NA	42.7	87.5	Multiple departments	66.7
Güler et al. (25)	Turkey	Cross-sectional	149	57.7	31	52.3	Intensive care unit	75.2
Isiek et al. (43)	Nigeria	Cross-sectional	250	66.4	38.7	82.0	Multiple departments	38.8
Jose et al. (44)	India	Cross-sectional	137	67.9	30.4	53.3	Multiple departments	30.7
Jung and Lee (45)	Korea	Cross-sectional	660	15	27.5	98	Multiple departments	79
Kandemir et al. (28)	Turkey	Cross-sectional	194	37.6	30	71.1	Intensive care unit	75.8
Kang et al. (46)	Korea	Cohort	287	NA	24	NA	Multiple departments	35.9
Lee and Choi (47)	Korea	Cross-sectional	234	39.4	33.4	87.2	Multiple departments	60.7
Lee et al. (24)	Korea	Cross-sectional	493	2.4	NA	94.7	Multiple departments	16.4
Li et al. (48)	China	Cross-sectional	1,204	52.8	30.4	91.2	Multiple departments	48.8
Li et al. (49)	China	Cohort	926	NA	33.4	80.7	Multiple departments	56
Liu et al. (50)	China	Cross-sectional	984	NA	NA	NA	Multiple departments	40.1
Luo et al. (51)	China	Cross-sectional	3,378	NA	NA	NA	Multiple departments	37.1
Mao et al. (16)	China	Cross-sectional	740	63.9	30.5	100	Multiple departments	8.2
Mehra et al. (17)	India	Cross-sectional	117	NA	39.8	84.6	Outpatient department	73.5
Melchor et al. (52)	China	Cross-sectional	196	51	42.9	80.1	Multiple departments	77.6
Mensinger et al. (53)	United States	Cross-sectional	467	54.8	38.1	91.9	Multiple departments	68.3
Nashwan et al. (54)	Qatar	Cross-sectional	200	80.0	NA	40.5	Multiple departments	76
Noh et al. (55)	Korea	Cross-sectional	161	7.5	27.6	88.8	Multiple departments	59.6
Oosterhuis-Nienhaus et al. (56)	Netherlands	Cross-sectional	722	NA	46.9	37.4	Emergency department	39.2
Peng et al. (31)	China	Cross-sectional	4,188	68.4	30	99.5	Multiple departments	54.6
Rahman et al. (57)	Bangladesh	Cross-sectional	154	NA	NA	NA	Multiple departments	13.6
Sadeghniiat-Haghighi et al. (58)	Iran	Cross-sectional	304	55	35	81.3	Multiple departments	79.9
Sagherian et al. (33)	United States	Cross-sectional	2,488	65.4	41.5	89.7	Multiple departments	42.2
Şahin et al. (59)	Turkey	Cross-sectional	254	65.7	NA	100.0	Multiple departments	60.6
Son and Ham. (60)	Korea	Cross-sectional	438	46.6	33.1	100	Multiple departments	50.5
Stocchetti et al. (18)	Italy	Cross-sectional	84	NA	38.5	63.4	Intensive care unit	70.2
Subih et al. (18)	Jordan	Cross-sectional	182	31.3	26.3	57.1	Intensive care unit	98.9
T. R et al. (61)	India	Cross-sectional	117	NA	NA	100	Multiple departments	45.3
Tiete et al. (62)	Belgium	Cross-sectional	468	NA	NA	NA	Multiple departments	72.6
Tuan et al. (34)	Vietnam	Cross-sectional	326	75.2	31.6	92.9	Multiple departments	36.2
Vitale et al. (30)	Italy	Cross-sectional	291	NA	NA	72.9	Intensive care unit	77.7
Vitale et al. (63)	Italy	Cross-sectional	408	NA	NA	75.2	Multiple departments	70.3
Wang et al. (64)	China	Cross-sectional	501	NA	NA	93.2	Multiple departments	51.3
Wen et al. (65)	China	Cross-sectional	552	54.4	28.6	97.5	Multiple departments	27.7
Witkoski Stimpfel et al. (66)	United States	Cross-sectional	629	64.7	42.9	92.3	Multiple departments	54.8
Xiao et al. (67)	China	Cross-sectional	1,766	56.6	NA	95.9	Multiple departments	76.8
Ye et al. (27)	China	Cross-sectional	437	55.2	32.2	92.0	Multiple departments	54
Zhan et al. (68)	China	Cross-sectional	1,874	70	33.7	85	Multiple departments	57.8
Zhang et al. (19)	China	Cross-sectional	200	72	32.1	88	Multiple departments	35
Zhang et al. (19)	China	Cross-sectional	1,630	70.5	33.2	93.6	Hemodialysis center	31.8

NA, Not available.

### 3.2 Assessment results of risk of bias

Based on the JBI criteria, 43 (70.5%) were rated as having a low risk of bias, and the remaining 8 (29.5%) were classified as moderate risk, with no study rated as high risk. At the domain level, an appropriate sample frame was identified in all studies, and a suitable sampling method was employed in 13 studies (25.5%). Sample size justification was adequate in 41 studies (80.4%) and unclear in 10 studies (19.6%). All studies met the criteria for a clear definition of study subjects and settings, adequate sample coverage, valid identification methods, standardized and reliable measurement of the condition, and appropriate statistical analysis. The adequacy of the response rate was confirmed in 19 studies (37.3%), deemed inadequate in 32 studies (62.7%), and remained unclear in 5 studies (9.8%).

### 3.3 Pooled prevalence of insomnia in nurses

**Figure 2:**
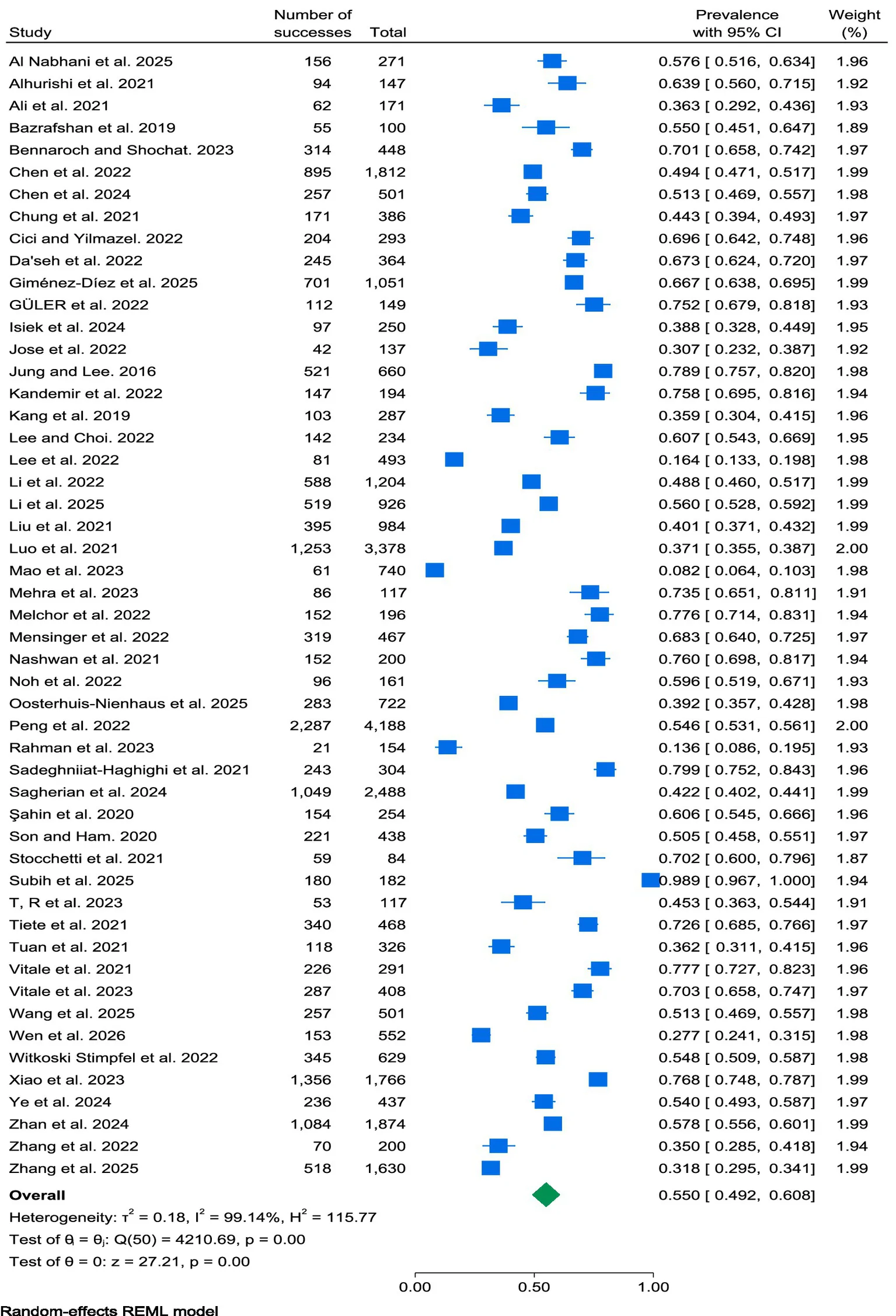
Forest plot of pooled prevalence of insomnia in nurses.

Given the considerable heterogeneity observed (*I*^2^ = 99.1%), a random-effects model was employed to pool the data. The overall prevalence of insomnia in nurses was estimated at 55.0% (95% CI: 49.2%–60.8%, 95% PI: 15.8%–90.8%) (Figure 2).

### 3.4 Subgroup analysis and meta-regression analysis

The results of subgroup analyses are detailed in Table 2 and Supplementary Figures S1–S4. Stratified by economic status, the pooled prevalence of insomnia was 58.9% (95% CI: 51.3–66.3%, I^2^ = 98.3%) in high-income ountries, 53.2% (95% CI: 44.9–61.4%, I^2^ = 99.3%) in upper-middle-income countries, and 50.6% (95% CI: 30.4–70.6%, I^2^ = 98.7%) in low-middle-income countries. However, the difference across economic groups was not statistically significant (*p* = 0.53). Regarding geographic region, the prevalence in Asia was 54.1% (95% CI: 47.2–61.0%, I^2^ = 99.2%), whereas the estimate for non-Asian regions was 5.2% (95% CI: 48.6–67.5%, I^2^ = 98.3%), with no significant between-group difference (*p* = 0.50). When analyzed by data collection period, studies conducted during the non-COVID-19 pandemic period yielded a prevalence of 62.2% (95% CI: 48.5–75.1%, I^2^ = 98.8%), compared to 52.8% (95% CI: 46.5–59.0%, I^2^ = 99.1%) for those conducted during the COVID-19 outbreak, with no significant difference between the two periods (*p* = 0.22). Subgroup analysis by work unit revealed that studies focusing on intensive care unit nurses reported a significantly higher prevalence (81.7, 95% CI: 67.4–92.6%, I^2^ = 95.9%) than those conducted in other units (51.9, 95% CI: 46.4–57.4%, I^2^ = 99.0%), with a significant between-group difference (*p* < 0.001).

**Table 2:** The results of subgroup analyses.

Subgroups	Number of studies	Prevalence (95% CI)	Heterogeneity		*p* values across subgroups
			*I* ^2^	*p* values	
*Economic condition*					0.53
High income	20	58.9% (51.3–66.3%)	98.3%	*p* < 0.001	
Upper-middle income	22	53.2% (44.9–61.4%)	99.3%	*p* < 0.001	
Low-middle income	9	50.6% (30.4–70.6%)	98.7%	*p* < 0.001	
*Geographic region*					0.50
Asia	40	54.1% (47.2–61.0%)	99.2%	*p* < 0.001	
Non-Asia	11	5.2% (48.6–67.5%)	99.1%	*p* < 0.001	
*Data collection period*					0.22
Non-COVID-19 pandemic period	12	62.2% (48.5–75.1%)	98.8%	*p* < 0.001	
During COVID-19 pandemic outbreak	39	52.8% (46.5–59.0%)	99.1%	*p* < 0.001	
*Work unit*					<0.001
Intensive care unit	5	81.7% (67.4–92.6%)	95.9%	*p* < 0.001	
Other unit	46	51.9% (46.4–57.4%)	99.0%	*p* < 0.001	

Univariate meta-regression (Table 3) showed that work unit (*p* < 0.001, R^2^ = 19.09) was a significant moderator of insomnia prevalence, whereas sample size, mean age, proportion of married participants, proportion of female participants, economic condition, geographic region, and data collection period were not (all *p* > 0.05).

**Table 3:** Meta-regression results of of the pooled prevalence of insomnia in nurses.

Variables	Number of studies	Coefficient	Standard Error	95% CI	t values	*p* values	R^2^ (%)
Sample size	51	−7.66 × 10^−5^	7.15 × 10^−5^	(−0.0002, 0.0000)	−1.66	0.284	0.27
Mean age	36	0.0032	0.0133	(−0.0229, 0.0294)	0.24	0.809	0
Proportion of married	35	−0.0039	0.009	(−0.0115, 0.0037)	−1.00	0.320	0
Proportion of females	44	−0.0071	0.0037	(−0.0143, 0.0001)	−1.92	0.053	6.11
*Economic condition (Ref: High income)*							0
Upper-middle income	22	−0.1153	0.1316	(−0.3731, 0.1426)	−0.88	0.381	
Low-middle income	9	−0.1679	0.1719	(−0.5048, 0.1690)	−0.98	0.329	
*Geographic region (Ref: Asia)*							0
Non-Asia	11	0.0824	0.1450	(−0.2017, 0.3665)	0.57	0.570	
*Data collection period (ref: Non-COVID-19 pandemic period)*							0
During COVID-19 pandemic outbreak	39	0.1910	0.1385	(−0.0804, 0.4625)	1.38	0.168	
*Work unit (ref: Intensive care unit)*							19.09
Other unit	46	−0.6433	0.1813	(−0.9986, −0.2880)	−3.55	<0.001	

### 3.5 Publication bias and sensitivity analysis

**Figure 3:**
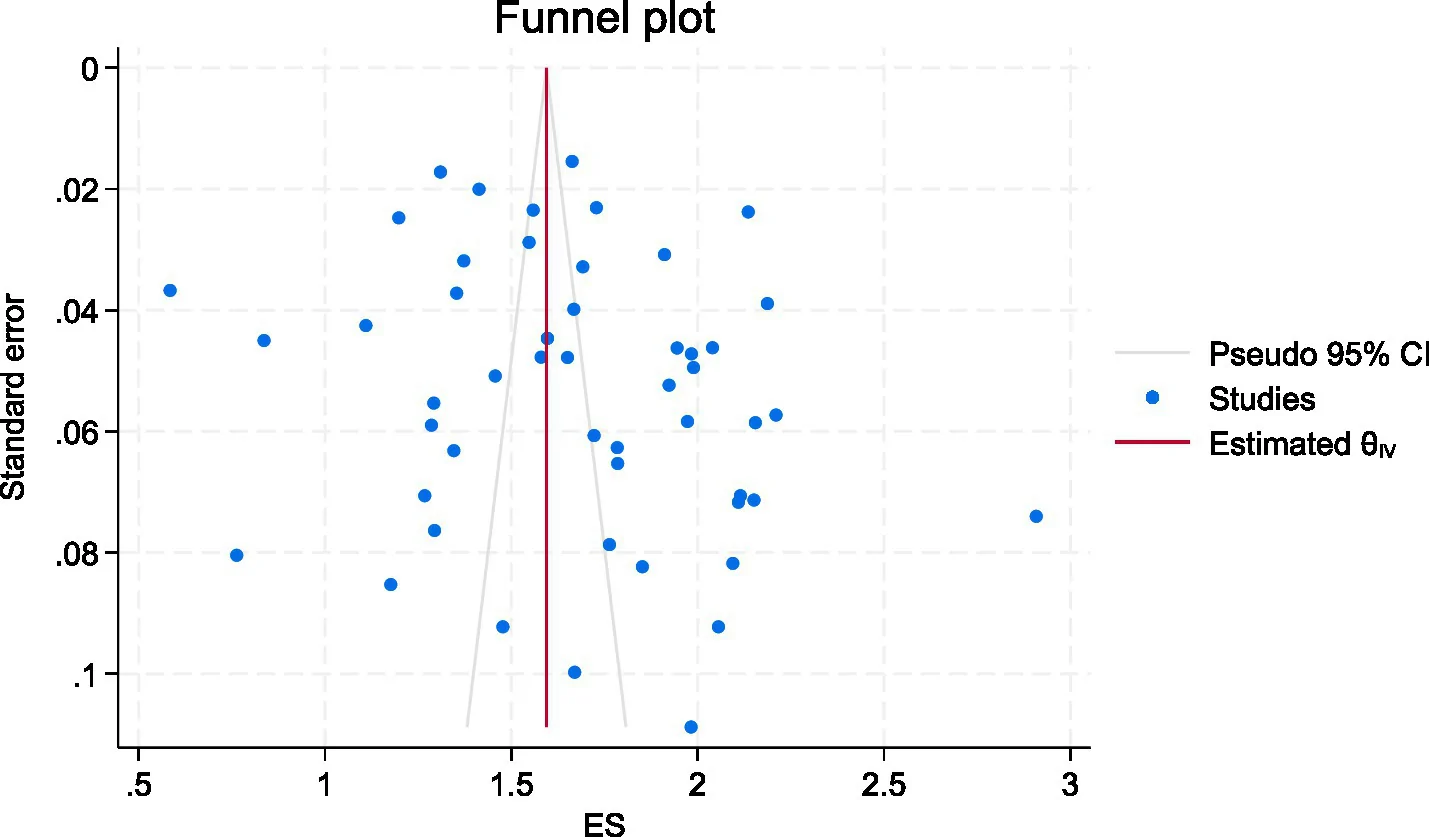
Funnel plot.

The funnel plot appeared roughly symmetrical (Figure 3), and Egger’s regression test further confirmed the absence of significant publication bias (Z = 1.36, *p* = 0.175; Supplementary Figure S5). The robustness of the pooled prevalence was assessed via leave-one-out sensitivity analysis (Supplementary Figure S6). Sequential exclusion of each study yielded pooled estimates ranging from 54.5% to 56.1%, with no individual study significantly altering the overall estimate.

## 4 Discussion

This meta-analysis aimed to estimate the pooled prevalence of insomnia and determine its potential moderating factors in nurses. The pooled analysis, which included 51 studies across 19 countries, revealed an overall insomnia prevalence of 55.0% among 36,971 nurses. However, given the substantial heterogeneity (high I^2^ statistic, wide confidence and prediction intervals), these findings should be interpreted with caution in nursing management practice. The substantial insomnia burden observed in nurses may be attributable to the synergistic effects of occupational attributes, circadian misalignment, psychosocial stress, and contextual environmental factors. Night shift work has been acknowledged as a significant risk factor for insomnia among nursing staff (7). Providing direct clinical care to patients is the primary duty of nurses, which generally requires them to be on duty around the clock and inevitably involves night shift rotations (24). This disrupts their circadian rhythms, making it difficult for them to recover deep sleep even on days off (18). Beyond the direct physiological disruption caused by circadian misalignment, behavioral and environmental factors may further compound the problem. In some cultural contexts, nursing staff may utilize post-night-shift free time for social activities, inadvertently delaying sleep onset and reducing total sleep opportunity, which undermines sleep consolidation and restorative efficiency (25). Moreover, daytime sleep, which constitutes the primary recovery period for night shift workers, frequently occurs under suboptimal environmental conditions, with household noise, ambient light, and social obligations at peak levels (26). These conditions lead to fragmented sleep architecture and impaired physiological recovery, failing to provide the restorative function necessary to counteract sleep debt accumulated during night shifts. Furthermore, nurses are frequently exposed to critical patient conditions and emergency procedures, which keep them in a state of sustained hypervigilance that persists after work, making it difficult to fall asleep (27). Meanwhile, prolonged exposure to suffering and death leads to emotional exhaustion that activates the sympathetic nervous system and suppresses melatonin secretion (8). These findings highlight an urgent need for organizational policies that prioritize insomnia prevention and management, particularly through the optimization of shift rotation, the provision of adequate rest periods, and the creation of sleep-friendly work environments.

In this meta-analysis, subgroup analyses and meta-regression both revealed that work unit was significantly associated with the pooled prevalence of insomnia among nurses. Specifically, nurses recruited from intensive care units exhibited a significantly higher prevalence of insomnia than those derived from mixed samples comprising various wards. The significantly higher prevalence of insomnia observed in nurses working in intensive care units may be explained by several occupational characteristics unique to this specialty. First, the intensive care unit environment is characterized by continuous noise from monitoring equipment, unpredictable alarm signals, and persistent lighting, all of which disrupt circadian rhythms and heighten physiological arousal (25). Second, intensive care unit nurses are routinely exposed to life-threatening patient conditions, emergency resuscitation, and mortality, which impose profound emotional and psychological burdens (28). The requirement for hypervigilance during shifts and the difficulty in psychologically detaching from work afterward further impair sleep initiation and maintenance. Third, the high-stakes decision-making environment, coupled with reduced autonomy and intense responsibility, contributes to chronic stress and emotional exhaustion (29). Finally, unfavorable shift schedules and insufficient recovery time are also recognized as primary contributors, rendering intensive care unit nurses particularly susceptible to insomnia (30). Beyond these specialty-specific characteristics, broader organizational and structural factors may further exacerbate insomnia risk among ICU nurses. For instance, institutional policies that maintain extended shift schedules, such as 12-h day shifts and 12-h night shifts, may severely limit recovery time and disrupt circadian rhythms more profoundly than shorter shift patterns (31). Similarly, inadequate nurse-to-patient ratios remain prevalent in many ICU settings, imposing excessive workload and increasing physical and mental fatigue, both of which are established contributors to sleep disturbances (32). Furthermore, insufficient rest breaks during shifts, limited access to on-site recovery facilities, and a lack of organizational support for sleep health may compound the problem. These organizational and structural factors, when combined with the inherent demands of ICU nursing, may create a particularly high-risk environment for insomnia (33). Therefore, intensive care unit nurses, who exhibit a significantly high risk of insomnia, should be given particular priority in the development and implementation of targeted organizational strategies by healthcare administrators, including optimized shift scheduling, protected rest periods during duty hours, and accessible psychological support services.

Our study demonstrated that sample size, mean age, proportion of married participants, proportion of female participants, economic condition, geographic region, and data collection period all did not exert significant moderating effects on the prevalence of insomnia among nurses. These findings suggest that the high prevalence of insomnia among nurses appears to be a pervasive occupational health issue that transcends geographical, economic, and demographic boundaries, and is not substantially altered by the COVID-19 pandemic context at the aggregate level. The non-significant moderating effects may be attributed to the universally high occupational stressors inherent to the nursing profession, such as shift work, high workload, emotional labor, and workplace violence, which operate consistently across diverse settings and time periods (2). In particular, the non-significant difference between non-COVID-19 and COVID-19 periods, though counterintuitive at first glance, may reflect the complex interplay of multiple factors. While the pandemic introduced additional stressors, it also brought increased institutional support, enhanced public recognition, and strengthened protective measures, which may have partially offset the negative psychological impact (34). However, given the substantial heterogeneity within subgroups and the limited number of studies in certain categories, these null findings should be interpreted with caution, and future studies with more granular data are warranted to further explore potential moderators.

### 4.1 Limitations

This study has several limitations. First, although no significant publication bias was detected, the restriction to English-language articles and the omission of unpublished studies and grey literature may have introduced such bias, potentially leading to overestimation of the pooled prevalence. Second, the majority of the included studies were conducted in Asia, which may limit the generalizability of our findings to other geographic regions with different cultural backgrounds, healthcare systems, and occupational environments. Third, in line with prior prevalence meta-analyses, considerable heterogeneity and broad confidence and prediction intervals were observed. However, work unit, the sole moderator examined, accounted for only a limited proportion of this between-study variance. Therefore, this pooled data should be interpreted not as a fixed population parameter but as a crude central tendency across disparate contexts. The wide prediction intervals further caution that true prevalence in any specific nursing setting may deviate substantially from this point estimate. These considerations collectively urge nursing administrators to contextualize the estimate within local workforce demographics and occupational conditions rather than applying it as a universal benchmark. Finally, all included studies assessed insomnia using the ISI. Although this instrument is well-validated and widely used in insomnia research, its reliance on self-report makes it inherently susceptible to recall and social desirability biases, which may have distorted prevalence estimates in either direction.

## 5 Conclusion

Our pooled estimates indicate that more than half of the nurses across the included studies were affected by insomnia. However, substantial heterogeneity and wide prediction intervals persisted, and only work unit and risk of bias partially explained this variation. These findings should therefore be interpreted with caution when translated into nursing management practice or policy recommendations. Furthermore, given the high burden of insomnia in this vulnerable occupational group, healthcare administrators should prioritize routine sleep screening and implement targeted interventions to alleviate insomnia among nurses.

## Data Availability

The datasets presented in this study can be found in online repositories. The names of the repository/repositories and accession number(s) can be found in the article/Supplementary material.
