# Supplementary material for: Prevalence of insomnia in nurses: a meta-analysis of observational studies using the insomnia severity index
Source: Front Public Health. 2026 Aug 12;14:1913239. doi: 10.3389/fpubh.2026.1913239 (PMC13506856; doi:10.3389/fpubh.2026.1913239)
Supplement: Supplementary file 1 [file Supplementary_file_1.docx]

**Supplemental Materials**

Table S1. The search strategies.

| Databases | Step | Search Strategies |
| --- | --- | --- |
| PubMed | #1 | "nurses"[Mesh Terms] Sort by: Best match |
|  | #2 | "nursing staff"[Mesh Terms] Sort by: Best match |
|  | #3 | "nurses"[Title/Abstract] OR "nursing staff"[Title/Abstract] OR "registered nurses"[Title/Abstract] OR "clinical nurses"[Title/Abstract] Sort by: Best match |
|  | #4 | "Sleep Initiation and Maintenance Disorders"[Mesh] Sort by: Best match |
|  | #5 | "insomnia"[Title/Abstract] OR "sleep disorder"[Title/Abstract] OR "sleeplessness"[Title/Abstract] OR "sleep disturbance"[Title/Abstract] Sort by: Best match |
|  | #6 | "Insomnia Severity Index"[Title/Abstract] OR "ISI"[Title/Abstract] Sort by: Best match |
|  | #7 | #1 OR #2 OR #3 Sort by: Best match |
|  | #8 | #4 OR #5 Sort by: Best match |
|  | #9 | #6 AND #7 AND #8 Sort by: Best match |
| Web of Science | #1 | TS=(nurses) OR TS=(nursing staff) OR TS=(registered nurses) OR TS=(clinical nurses) |
|  | #2 | TS=(insomnia) OR TS=(sleep disorder) OR TS=(sleeplessness) OR TS=(sleep disturbance) |
|  | #3 | TS=(Insomnia Severity Index) OR TS=(ISI) |
|  | #4 | #1 AND #2 AND #3 |
| Scopus | #1 | ABS("[nurses](https://www.ncbi.nlm.nih.gov/mesh/68009369)" OR "nursing staff" OR "registered nurses" OR "clinical nurses") |
|  | #2 | ABS("insomnia" OR "sleep disorder" OR sleeplessness OR "sleep disturbance") |
|  | #3 | ABS("Insomnia Severity Index" OR "ISI") |
|  | #4 | #1 AND #2 AND #3 |
| Embase | **#1** | '[nurses](https://www.ncbi.nlm.nih.gov/mesh/68009369)'/exp |
|  | **#2** | 'nursing staff'/exp |
|  | **#3** | 'nurses':ti,ab,kw OR 'nursing staff':ti,ab,kw OR 'registered nurses':ti,ab,kw OR 'clinical nurses':ti,ab,kw |
|  | **#4** | 'insomnia'/exp |
|  | #5 | sleep disorder'/exp |
|  | **#6** | **'insomnia':ti,ab,kw OR 'sleep disorder':ti,ab,kw OR sleeplessness:ti,ab,kw OR 'sleep disturbance':ti,ab,kw** |
|  | **#7** | **'Insomnia Severity Index':ti,ab,kw OR ISI:ti,ab,kw** |
|  | **#8** | #1 OR #2 OR #3 |
|  | **#9** | #4 OR #5 OR #6 |
|  | **#10** | #7 AND #8 AND #9 |
| Cochrane Library | #1 | **MeSH descriptor: [Nursing staff] explode all trees** |
|  | #2 | (nurses):ti,ab,kw OR (nursing staff):ti,ab,kw OR (registered nurses):ti,ab,kw OR (clinical nurses):ti,ab,kw |
|  | #3 | MeSH descriptor: [Sleep Initiation and Maintenance Disorders] explode all trees |
|  | #4 | (insomnia):ti,ab,kw OR (sleep disorder):ti,ab,kw OR (sleeplessness):ti,ab,kw OR (sleep disturbance):ti,ab,kw |
|  | #5 | (Insomnia Severity Index):ti,ab,kw OR (ISI):ti,ab,kw |
|  | #6 | #1 OR #2 |
|  | #7 | #3 OR #4 |
|  | #8 | #5 AND #6 AND #7 |
| CINAHL | #1 | MH [nurses](https://www.ncbi.nlm.nih.gov/mesh/68009369) OR MH nursing staff OR MH registered nurses OR MH clinical nurses |
|  | #2 | **MH insomnia OR MH sleep disorder OR MH** sleeplessness **OR MH** sleep disturbance |
|  | #3 | MH Insomnia Severity Index OR MH ISI |
|  | #4 | #1 AND #2 AND #3 |
| PsycINFO | #1 | MH [nurses](https://www.ncbi.nlm.nih.gov/mesh/68009369) OR MH nursing staff OR MH registered nurses OR MH clinical nurses |
|  | #2 | **MH insomnia OR MH sleep disorder OR MH** sleeplessness **OR MH** sleep disturbance |
|  | #3 | MH Insomnia Severity Index OR MH ISI |
|  | #4 | #1 AND #2 AND #3 |

Table S2. Quality assessment results of included studies.

| Study | Q1 | Q2 | Q3 | Q4 | Q5 | Q6 | Q7 | Q8 | Q9 | Overall |
| --- | --- | --- | --- | --- | --- | --- | --- | --- | --- | --- |
| Al Nabhani et al. 2025 | Y | Y | Y | Y | Y | Y | Y | Y | Y | L |
| Alhurishi et al. 2021 | Y | N | U | Y | Y | Y | Y | Y | U | M |
| Ali et al. 2021 | Y | N | Y | Y | Y | Y | Y | Y | U | L |
| Bazrafshan et al. 2019 | Y | Y | U | N | Y | Y | Y | Y | U | M |
| Bennaroch and Shochat. 2023 | Y | N | Y | Y | Y | Y | Y | Y | U | L |
| Chen et al. 2022 | Y | N | Y | Y | Y | Y | Y | Y | Y | L |
| Chen et al. 2024 | Y | N | Y | Y | Y | Y | Y | Y | Y | L |
| Chung et al. 2021 | Y | N | Y | Y | Y | Y | Y | Y | U | L |
| Cici and Yilmazel. 2022 | Y | Y | U | U | Y | Y | Y | Y | U | M |
| Da'seh et al. 2022 | Y | N | Y | Y | Y | Y | Y | Y | U | L |
| Giménez-Díez et al. 2025 | Y | N | U | Y | Y | Y | Y | Y | U | M |
| GÜLER et al. 2022 | Y | N | U | Y | Y | Y | Y | Y | U | M |
| Isiek et al. 2024 | Y | Y | Y | Y | Y | Y | Y | Y | U | L |
| Jose et al. 2022 | Y | Y | U | Y | Y | Y | Y | Y | U | L |
| Jung and Lee. 2015 | Y | Y | Y | Y | Y | Y | Y | Y | Y | L |
| Kandemir et al. 2022 | Y | N | Y | Y | Y | Y | Y | Y | U | L |
| Kang et al. 2019 | Y | Y | Y | Y | Y | Y | Y | Y | Y | L |
| Lee and Choi. 2022 | Y | N | Y | Y | Y | Y | Y | Y | U | L |
| Lee et al. 2022 | Y | N | Y | Y | Y | Y | Y | Y | U | L |
| Li et al. 2022 | Y | N | Y | Y | Y | Y | Y | Y | Y | L |
| Li et al. 2025 | Y | N | Y | Y | Y | Y | Y | Y | Y | L |
| Liu et al. 2021 | Y | Y | Y | Y | Y | Y | Y | Y | Y | L |
| Luo et al. 2021 | Y | Y | Y | Y | Y | Y | Y | Y | Y | L |
| Mao et al. 2023 | Y | N | Y | Y | Y | Y | Y | Y | U | L |
| Mehra et al. 2023 | Y | N | U | Y | Y | Y | Y | Y | U | M |
| Melchor et al. 2022 | Y | N | Y | Y | Y | Y | Y | Y | U | L |
| Mensinger et al. 2022 | Y | N | Y | Y | Y | Y | Y | Y | U | L |
| Nashwan et al. 2021 | Y | N | U | Y | Y | Y | Y | Y | U | M |
| Noh et al. 2022 | Y | N | Y | Y | Y | Y | Y | Y | Y | L |
| Oosterhuis-Nienhaus et al. 2025 | Y | Y | Y | Y | Y | Y | Y | Y | Y | L |
| Peng et al. 2022 | Y | N | Y | Y | Y | Y | Y | Y | Y | L |
| Rahman et al. 2023 | Y | N | Y | Y | Y | Y | Y | Y | U | L |
| Sadeghniiat-Haghighi et al. 2021 | Y | Y | Y | Y | Y | Y | Y | Y | Y | L |
| Sagherian et al. 2024 | Y | Y | Y | Y | Y | Y | Y | Y | U | L |
| Şahin et al. 2020 | Y | N | Y | Y | Y | Y | Y | Y | U | L |
| Son and Ham. 2020 | Y | N | Y | Y | Y | Y | Y | Y | U | L |
| Stocchetti et al. 2021 | Y | N | U | Y | Y | Y | Y | Y | Y | L |
| Subih et al. 2025 | Y | N | Y | Y | Y | Y | Y | Y | U | L |
| T, R et al. 2023 | Y | N | Y | Y | Y | Y | Y | Y | U | L |
| Tiete et al. 2021 | Y | N | Y | Y | Y | Y | Y | Y | U | L |
| Tuan et al. 2021 | Y | N | Y | Y | Y | Y | Y | Y | Y | L |
| Vitale et al. 2021 | Y | N | U | Y | Y | Y | Y | Y | U | M |
| Vitale et al. 2023 | Y | N | Y | Y | Y | Y | Y | Y | U | L |
| Wang et al. 2025 | Y | Y | Y | Y | Y | Y | Y | Y | Y | L |
| Wen et al. 2026 | Y | N | Y | Y | Y | Y | Y | Y | U | L |
| Witkoski Stimpfel et al. 2022 | Y | N | Y | Y | Y | Y | Y | Y | U | L |
| Xiao et al. 2023 | Y | N | Y | Y | Y | Y | Y | Y | Y | L |
| Ye et al. 2024 | Y | N | Y | Y | Y | Y | Y | Y | U | L |
| Zhan et al. 2024 | Y | N | Y | Y | Y | Y | Y | Y | Y | L |
| Zhang et al. 2022 | Y | N | Y | Y | Y | Y | Y | Y | Y | L |
| Zhang et al. 2025 | Y | N | Y | Y | Y | Y | Y | Y | U | L |

Notes: Y: Yes, N: No, U: Unclear. L: Low risk, M: Moderate risk, H: High risk.

Q1: Was the sample frame appropriate to address the target population?

Q2: Were study participants sampled in an appropriate way?

Q3: Was the sample size adequate?

Q4: Were the study subjects and the setting described in detail?

Q5: Was the data analysis conducted with sufficient coverage of the identified sample?

Q6: Were valid methods used for the identification of the condition?

Q7: Was the condition measured in a standard, reliable way for all participants?

Q8: Was there appropriate statistical analysis?

Q9: Was the response rate adequate, and if not, was the low response rate managed appropriately?

Figure S1. The pooled prevalence of insomnia in nurses based on economic condition.

Figure S2. The pooled prevalence of insomnia in nurses based on geographic region.

Figure S3. The pooled prevalence of insomnia in nurses based on data collection period.

Figure S4. The pooled prevalence of insomnia in nurses based on work unit.

Figure S5. Egger's test results.

Figure S6. Leave-one-out sensitivity analysis.
